# The Portuguese NHS 2024 reform: transformation through vertical integration

**DOI:** 10.3389/fpubh.2024.1389057

**Published:** 2024-05-23

**Authors:** Francisco Goiana-da-Silva, Juliana Sá, Miguel Cabral, Raisa Guedes, Rafael Vasconcelos, João Sarmento, Alexandre Morais Nunes, Rita Moreira, Marisa Miraldo, Hutan Ashrafian, Ara Darzi, Fernando Araújo

**Affiliations:** ^1^Centre for Health Policy, Institute of Global Health Innovation, Imperial College London, London, United Kingdom; ^2^NOVA Medical School, Universidade NOVA de Lisboa, Lisbon, Portugal; ^3^Faculdade de Ciências da Saúde, Universidade da Beira Interior, Covilhã, Portugal; ^4^Portuguese National Health Service Executive Board, Porto, Portugal; ^5^ULS Santo António, Porto, Portugal; ^6^ULS São João, Porto, Portugal; ^7^ULS Região de Leiria, Leiria, Portugal; ^8^Public Health Research Center, NOVA National School of Public Health, Universidade NOVA de Lisboa, Lisbon, Portugal; ^9^Centre for Public Administration and Public Policies, Institute of Social and Political Sciences, University of Lisbon, Lisbon, Portugal; ^10^Department of Management, Centre for Health Economics & Policy Innovation (CHEPI), Imperial College Business School, London, United Kingdom; ^11^Department of Surgery and Cancer, Faculty of Medicine, Imperial College London, London, United Kingdom; ^12^Faculty of Medicine, University of Porto, Porto, Portugal

**Keywords:** integrated care, service innovation, health outcomes, vertical integration, Portuguese NHS, healthcare reform

## Abstract

Vertical integration models aim for the integration of services from different levels of care (e.g., primary, and secondary care) with the objective of increasing coordination and continuity of care as well as improving efficiency, quality, and access outcomes. This paper provides a view of the Portuguese National Health Service (NHS) healthcare providers’ vertical integration, operationalized by the Portuguese NHS Executive Board during 2023 and 2024. This paper also aims to contribute to the discussion regarding the opportunities and constraints posed by public healthcare organizations vertical integration reforms. The Portuguese NHS operationalized the development and generalization of Local Health Units management model throughout the country. The same institutions are now responsible for both the primary care and the hospital care provided by public services in each geographic area, in an integrated manner. This 2024 reform also changed the NHS organic and organizational structures, opening paths to streamline the continuum of care. However, it will be important to ensure adequate monitoring and support, with the participation of healthcare services as well as community structures and other stakeholders, to promote an effective integration of care.

## 1 Introduction

Managing healthcare is challenging. In most European countries, the national health systems have several complexities, bureaucratic idiosyncrasies, continuous organizational and technological growth, as well as a long history spanning over 70 years (1). Organizations, such as national health services, need to be restructured when they undergo significant size changes, especially as they reach over 40 years of existence, which is precisely the stage most are reaching. According to Drucker, when an organization exhibits these characteristics, “it has outgrown its policies and its rules of behavior. If it continues in its old ways, it becomes ungovernable, unmanageable, uncontrollable” (2).

Integration of care is emerging as a tool for health sector reform around the world (3–7) to improve system efficiency and effectiveness, population health outcomes, continuity of care, as well as access to healthcare, both primary and secondary (3–7).

There are several integrated care models, and their rationale and value vary according to its contexts. However, integrated care models can be comprehended as a set of structures and practices shaped by contextual factors. Their aim is to unify inputs, delivery, management, and organization of health services under a single management scope (3, 7–11). Often, it is also antithetical to fragmented and episodic care, which has also been dubbed “Healthcare Archipelago.” In its latter definitions, integration of care is associated to a people-center and user-led health system (8, 12).

The concept of integrated care primarily originates from systems theory, which views organizations as hierarchical structures with interconnected parts (7). As organizations become more complex and decentralized with specialization, quality of care may suffer. Pursuing the integration of these specialized structures can therefore lead to increased efficiency and healthcare quality (7, 13).

The gains in efficiency and effectiveness can occur in multiple aspects. Integration can lead to less duplication and waste, more flexible service provision, and better coordination and continuity of care. It can encourage more holistic and personalized approaches to health needs, especially for complex patients such as older individuals and those with multiple chronic diseases (4, 7).

Integrated care is also considered a strategy to increase continuity of care, with an emphasis on prevention and disease management. This is achieved by shifting the focus of care to the community and primary care, instead of the hospital, and even changing how accident and emergency departments in hospitals operate (14).

Specific areas present a compelling rationale for integration, such as the cases that demand synergies between social care and healthcare. Integration can significantly impact discharge times from hospitals and overall quality of care in specific cases, such as the ones related to mental illness, palliative care, older adults, and children. For example, integrating health and social care reduced the length of hospital stay by 32% in a study involving patients with serious mental illness (15).

Worldwide efforts are being made to increase the integration of care in different formats, ranging from complete integration of the organizations responsible for health and social care to small efforts in decentralized service-delivery networks (6).

Integrated care can take many different forms and dimensions and can be either vertical or horizontal. Horizontal integration applies to providers or groups operating at the same level (e.g., groups of primary health centers). Vertical integration involves bringing together different levels on the hierarchical structure under one management umbrella (e.g., secondary and community care) (4, 8) aiming to provide a continuum of primary, acute, and post-acute care within a single organizational structure. In vertical integration, the governance, planning, and resourcing of care are carried out by the same organization. This approach is expected to improve service coordination and facilitate a better progression of services across the patient care spectrum (6, 8, 16).

Supported by the previous experience with vertically integrated healthcare organizations in Portugal and best practices from the literature, the Portuguese National Health Services (NHS) Executive Board (EB) is implementing a novel approach in vertical integration. The purpose of this article is to present an overview of the literature and the efforts being done in the Portuguese NHS. Thus, the article aims to contribute to the global discussion on the practical implementation of integration of care, in the context of a health system strongly supported by a national health service.

## 2 Subjects of discussion

Integration of care has promising results in reducing length of hospital stays (4), enhancing patient satisfaction (4, 14), increasing perceived quality of care, and enabling access to services (14), both in European and global settings.

Despite that, the effectiveness of integrated care initiatives hinges on a multitude of factors. There is a need for a careful orchestration of the interconnected elements, each contributing to the overarching goal of enhancing healthcare delivery and improving patient outcomes. These factors include:

- The level and scope of integration, which determine the extent to which services are coordinated across various healthcare domains (13);
- Financial incentives that affect, directly or indirectly, integrated care models, which can either foster or hinder collaborative efforts among healthcare providers (13);
- The degree of shared decision-making among stakeholders, as it promotes patient-centered approaches and ensures that care plans align with individual needs and preferences (13, 17);
- The establishment of robust governance structures, which are essential for promoting cohesive collaboration and ensuring accountability across the care continuum (13, 18);
- The development of care paths to specific disease contexts, as different conditions may require tailored approaches to integration (13, 18).

## 3 Discussion

While integration efforts hold theoretical benefits, their implementation results vary in both benefits and expected disadvantages. Hence, focusing on specific contexts and implementation methods is crucial.

The United Kingdom took a variety of approaches to care integration and has managed to improve provider experience through more responsive services, as well as reduce costs per patient per year (6, 13, 19, 20). Furthermore, patient waiting time and outpatient appointments may be reduced, and patient needs and preferences with regards to end-of-life care are met more often (14). Scotland, specially, found that vertical integration of health and social care can enhance patient outcomes, organizational efficiency, and patient experience (6). It also reduced delayed discharges – patients clinically ready for discharge awaiting post-hospital care and support (6).

Germany’s *Gesundes Kinzigtal*, a population-based approach that organizes care across all health service sectors in a specific region has also achieved considerable results. A decrease in mortality was measured 2.5 years after enrollment in the program, as well as decreased length of stay, improved patient and provider experience, and decreased cost per patient per year (13).

In the Netherlands, a bundled payments system, specific for certain chronic conditions reduced specialist care and enhanced patient and provider experience. However, it presented mixed results for clinical outcomes, mortality, and process indicators and increased costs due to its disease-oriented approach (13).

In the US, despite its predominantly private healthcare system, there are numerous attempts to implement integration of care in its various forms. A study on medication adherence rates found no average changes, yet health equity worsened, with significant declines in adherence among minorities, patients over 80 years old, and with greater comorbidities. This was explained as a decrease competition for Medicare beneficiaries and disruptions of services among hospital-physician integration (21). Despite that, even in these circumstances vertical integration has shown some benefits. In a systematic review of vertical integration and quality of care in the US, 6 out of 10 studies found that vertical integration was associated with higher quality of care (22) and vertical integration was associated with increased access to surgical care for vulnerable, low-income patients (23).

In Spain, the integration of 1 hospital and 7 primary care providers, the provision of cardiology care improved the quality of care related to ischemic heart disease, heart failure, and atrial fibrillation, when compared to usual cardiological care (24).

Overall, vertical integration is a promising avenue to increase patient-centered care while still increasing systemic efficiency and effectiveness. Furthermore, attempts with greater range are more promising than focusing only on one disease or group (13). While care integration typically enhances results and quality in health, vigilance is required to mitigate potential inefficiencies, especially when strategic alignment is not promoted (25).

Several pitfalls of vertical integration warrant consideration. There is a risk of price escalation instead of reduction, especially in contexts where vertical integration reduces competitiveness or when services and goods outside of bundled payments are involved in vertical integration (13, 26). Furthermore, various system attributes and market factors can influence the outcomes of vertical integration efforts. Those include changes in physician or healthcare team composition, patients’ health plans, market consolidation levels, payment policies, healthcare technology, and organizational practices (26). Addressing these complexities is crucial for optimizing the outcomes of vertical integration initiatives and mitigating potential adverse effects.

### 3.1 Vertical integration of care in Portugal

#### 3.1.1 Brief history

Health successes in Portugal are recognized worldwide, but the country will face health related challenges of the greatest magnitude in the next few decades (22). There is a profound change in its demographic and epidemiological profile, with the aging of the population and the predominance of chronic non-communicable diseases. Migration movements, both to leave and to enter the country, will bring its own share of challenges that will have to be addressed by its NHS (22). Nonetheless, the current system continues to be an international reference for Universal Health Coverage. The latest data from the World Health Organization UHC Service Coverage Index puts Portugal in the 5th highest rank in the world, tied with Germany and the United Kingdom (27).

The diversity of care that the Portuguese NHS provides, the capillarity of its services, the highly technical autonomy of its health professionals, the increasing healthcare costs and the expectations of a more informed and demanding society are friction points that need to be addressed to create a stronger health system in Portugal. Therefore, it is essential to promote multidimensional integration. There is a need to qualify the response, simplify processes, and increase coordination between teams of health professionals. All with a focus on experience and pathways between the different levels of care experienced by users. This should include (i) creating greater proximity of institutions in the same geographic area, (ii) improving participation of citizens, communities, professionals, and local authorities, (iii) monitoring and evaluating health policies, and (iv) maximizing access and efficiency of the health service response.

The Portuguese NHS has its roots in the 1940s, with the beginning of a hospital network. In the 1960s, integration was already considered as a path to the future, not only for administrative structures but also encompassing medical careers, coordination, and health services (28). The 1974 political revolution led to the creation of the Portuguese NHS as a universal and tax-based system (29). Since then, the epidemiological and socioeconomic contexts have improved over the years. Nonetheless, challenges to ensure humanism, universality and proximity remain (27).

The growing healthcare demands and health workforce specialization led to a considerable fragmentation between healthcare institutions around the country. In 2023, in total, there were around a hundred institutions, both on the hospital and primary care level. These institutions include, for example, (i) conglomerates of hospitals under the same management, (ii) healthcare center groupings that in turn would include several kinds of primary care services such as family medicine, at home care, home palliative care, public health units, among others, (iii) Local Health Units that would include all the before mentioned organizations under one management team, (iv) as well as specialty oncology hospitals.

Portugal began its first attempts of vertical integration in healthcare in the late 1990s. A new integrated healthcare structure, bringing primary care units and hospitals from the same region under the same leadership, budget and goals was developed: the Local Health Units (LHU) (30). The aim behind this organizational innovation was “creating conditions that enable better management of its institutions and the best articulation of these institutions among themselves and with others” (31).

The LHUs are legal entities with administrative, financial, and patrimonial autonomy (32). Hence, their ultimate objective is to enhance the connection between primary healthcare and specialized care. This takes place by integrating the provision and management of all healthcare levels from a given geographical area, ensuring both are housed within the same institution (33).

**Figure 1:**
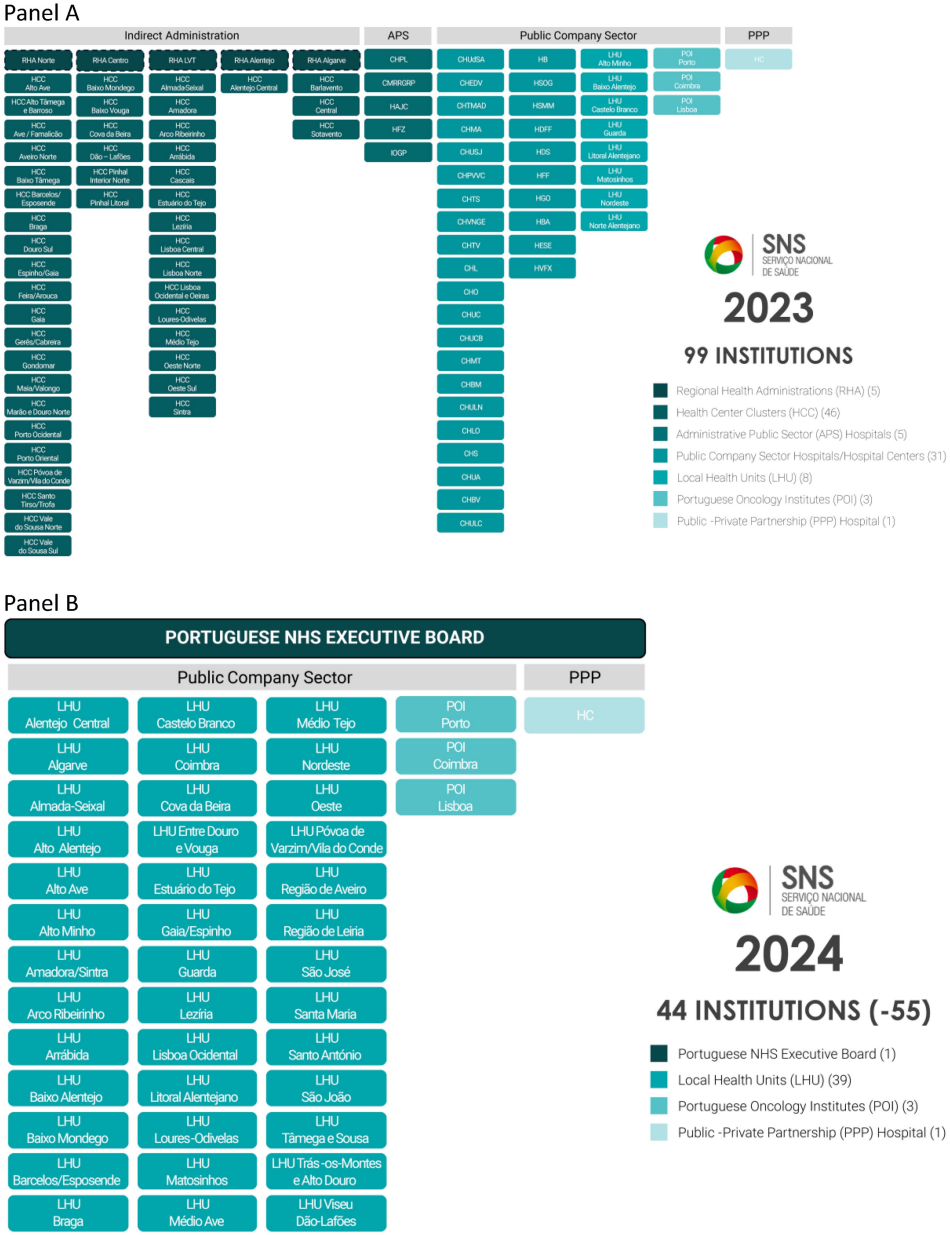
Organogram of the Portuguese National Health Service (SNS) institutions in 2023 **(Panel A)** and 2024 **(Panel B)**.

In 2022, a new statute of the NHS was approved. It created an executive board for the NHS, based on the need to simplify the health service organizational structure that, thus far, had been based in multiple vertical layers and institutions. It also aims to ensure that all the NHS operates as a network, a role that proved to be crucial in the fight against the COVID-19 pandemic (34). In August 2023, the government announced the expansion of the LHU model to the whole NHS for 2024, creating 31 new LHU in addition to the 8 already in operation. Before the current reform, LHUs provided care to a population of over 1 million inhabitants, around 10% of the national population (33). This implies the organization of the NHS into 39 LHU (30), apart from three oncology institutes (in Lisbon, Porto, and Coimbra) and the Cascais Hospital, managed as a public-private partnership (35). This will represent a streamline and flattening of the hierarchical structure that will now be comprised of 44 intuitions, less than half of the previous organization, as depicted in Figure 1.

At the national level, the goal is to optimize service responsiveness through streamlining the organizational structures of the Portuguese NHS, thereby presenting a clear and functional organizational framework (36). The mergers entailed in the reform were addressed through an intensive onboarding process of the institutions, that involved 27 working groups with weekly meetings, hospital management councils, clinical and health councils. Several of them went further and created participatory processes with the front-line workers to build from the bottom up what each LHU should be and how to organize itself. Indeed, the working groups that developed a business plan for their LHU were motivated by the NHS EB to tailor their plan to the specific characteristics of their population and their professionals.

#### 3.1.2 Future

The LHUs will redefine the organic architecture of the NHS institutions, assuming the assistance response at the level of primary health care and hospital care in an integrated manner. This reform considerably enhances and streamlines the construction of NHS instruments for planning and organization. Positive effects on health are expected, through the optimization and integration of care, proximity to care, management autonomy, reinforcement of primary health care, and focus on users. This reorganization will bring closer the point of care and the first level of an executive decision-making body. It will also decrease the average geographical scope that the first level of an executive decision-making body will have to care for, thus allowing for greater focus and better tailored management. Coordination with local authorities and the role of citizens will also be reinforced with this strategy.

The Portuguese perspective of integration of care through a single LHU system meets a concept of integration that focuses on the patient through two distinct models: structural integration that comprises the ownership of infrastructures and the services offered therein through an articulated supply chain, and functional integration that enhances effective service coordination (25).

Few studies identify areas for improvement in the existing LHUs. One of these considerations is that the financial model, until now, did not consider important sociodemographic characteristics, the distribution of diseases across different age groups, and the needs of the population (37). This is being tackled with a new financing model that proposes a population-based needs-driven approach, aiming for citizen-centered intervention. All while promoting the mitigation of avoidable acute episodes and associated hospitalizations. This logic values health promotion, disease prevention, early diagnosis, timely treatment, and appropriate rehabilitation (38). Another consideration is the need for better people-to-people integration, with focus on the user (39).

Nunes (40) conducted a SWOT analysis of this specified reform, while also considering other changes taking place in the overall healthcare system, identified six strengths and essential points of this measure (Table 1) and concluded that the model is promising. The key opportunities in the integration of healthcare services lie in strengthening primary care, maximizing resource efficiency, and promoting citizen participation. By focusing on people-centered care and empowering local authorities, healthcare systems can enhance effectiveness and adaptability. However, significant challenges such as the increasing health needs of the population and resistance to organizational change must be addressed to ensure successful integration efforts.

**Table 1:** SWOT analysis of the LHU model.

Strengths	Weaknesses
The establishment of effective coordination mechanisms among various levels of care. The proximity of provision that refers to the spatial closeness or nearness of a certain service or resource to its intended users or beneficiaries. The integration of primary healthcare and hospital care management, ensuring that beneficiaries of the SNS have equitable access to the most appropriate care based on their specific requirements. The acquisition of health benefits by means of proximity to decision-making processes and the attainment of enhanced autonomy. Advocate for the prioritization of primary health care as the fundamental pillar of the healthcare system Enhanced capabilities and independence of institutions through increased competencies	Insufficient readiness of infrastructures Challenges in achieving seamless integration of information systems Potential financial constraints, particularly for University Hospital Centers within the LHU framework The level of participation among municipalities exhibits asymmetry based on their political alignment. The potential for inadequate financial resources linked to performance incentives centered on outcomes and the generation of value. The new LHU model has a heightened level of diversity and complexity, deviating from the existing model of administration and supervisory bodies. The risk of failing to uphold the terms of authority delegation to local governing bodies The potential transfer of outstanding debts from defunct entities leading to the establishment of LHUs

Opportunities	Threats
Network management Strengthening health promotion and disease prevention policies Strengthening primary care in the proximity response Maximize access, quality and efficiency in resource management Meet the growing demands and expectations of citizens Participation of citizens, communities, professionals, and local authorities in the definition, monitoring, and evaluation of health policies Focus on people, that is, centering care on citizens Increased efficiency and effectiveness Reinforcement of necessary means and resources Greater management autonomy Stratification of the population by risk, which identifies the distribution of the disease burden in the population Promote differentiation, training, and research	Increased health and well-being needs of the population Aging Disease burden Organizational culture installed Lack of municipal collaboration The hospital-centric system installed in the perception of citizens, responsible for non-urgent access to emergency services Coordination between teams of healthcare professionals Transition of workers and healthcare professionals

Adapted from: Nunes (2024) (40).

The implementation of these measures requires a parallel process of close follow-up. The NHS EB is providing close support and townhall meetings with all leaders of the institutions of the NHS, as well as for specific areas of action, to share the current challenges and best practices during this change process. A thorough and open analysis of the clinical and financial impacts of this form of organization is essential. Therefore, the expansion of the LHU model, even if based on a careful assessment of the pros and cons and international scientific evidence, requires an independent assessment with a view to continuous improvement of the ongoing transformation. An independent public university will be conducting the evaluation process with benchmarking, quantitative, and qualitative data.

While this article serves as an initial discussion point regarding integration in the Portuguese NHS, there is a plethora of avenues for future research exploration. These include, but are not limited to: (i) examining patients’ perspectives on their experiences accessing primary, secondary, and emergency care services, (ii) analyzing outcome disparities among patients with multiple comorbidities pre-and post-vertical integration, (iii) assessing the impact of vertical integration on emergency attendances, admissions, and readmissions, as well as length of hospital stays, (iv) evaluating access to primary care, (v) investigating the capacity of vertical integration reforms to enhance recruitment and retention of healthcare professionals, and (vi) assessing financial results from the integration processes, among others. It also includes additionally, exploring the potential for economies of scale in the provision of back-office functions warrants further investigation (41).

## 4 Conclusion

The literature lacks reports of vertical integration as extensive as Portugal’s NHS-wide approach. Partial experiments show positive effects on efficiency, effectiveness, and health outcomes. Nonetheless, it is important to consider the risks inherent in the model and ensure adequate support in terms of promoting the real integration of care. A health system-wide approach is needed, which includes the participation of both the government and society. Currently, Portugal is deciding to take this step forward, supporting the mobilization and engagement of institutions, professionals, and communities looking to the goal of achieving better health for all.

## Data availability statement

The original contributions presented in the study are included in the article/supplementary material, further inquiries can be directed to the corresponding author.

## Author contributions

FG-d-S: Conceptualization, Writing – original draft, Writing – review & editing. JuS: Data curation, Investigation, Writing – original draft, Writing – review & editing. MC: Writing – original draft, Writing – review & editing. RG: Investigation, Writing – original draft, Writing – review & editing. RV: Investigation, Visualization, Writing – review & editing. JoS: Writing – review & editing. AM: Writing – review & editing. RM: Supervision, Writing – original draft, Writing – review & editing. MM: Supervision, Validation, Writing – review & editing, Writing – original draft. HA: Writing – original draft, Writing – review & editing. AD: Writing – original draft, Writing – review & editing. FA: Validation, Conceptualization, Funding acquisition, Investigation, Methodology, Project administration, Supervision, Writing – original draft, Writing – review & editing.
